# Characteristics, Risks, and Prevention of Rhegmatogenous Retinal Detachment in the Contralateral Eye

**DOI:** 10.3390/jcm14010222

**Published:** 2025-01-02

**Authors:** Rami Al-Dwairi, Omar Saleh, Hasan Mohidat, Seren Al Beiruti, Ali Alshami, Leen El Taani, Abdullah Sharayah, Ahmed H. Al Sharie, Abdelwahab Aleshawi

**Affiliations:** 1 Division of Ophthalmology, Department of Special Surgery, Faculty of Medicine, Jordan University of Science & Technology, Irbid 22110, Jordan; 2 Department of Pathology and Microbiology, Faculty of Medicine, Jordan University of Science & Technology, Irbid 22110, Jordan

**Keywords:** rhegmatogenous retinal detachment, retinopexy, lattice degradation, silicone oil

## Abstract

**Background/Objectives**: Rhegmatogenous retinal detachment (RRD) is a potentially blinding retinal disorder. RRD in the first eye is a well-recognized risk factor for bilateral RRD since risk factors that predispose to RRD affect both eyes. In this study, we assess the presenting factors that predispose individuals to bilateral RRD and evaluate the role of prophylactic retinopexy in preventing fellow-eye RRD. **Methods**: Retrospectively, all patients who underwent RRD repair through pars plana vitrectomy were included. A medical database was utilized to extract the data. The primary outcome was to report the development of RRD in the fellow eyes according to the presenting risk factors. Secondary outcomes included the prophylactic effect of laser retinopexy for the fellow eye. **Results**: In this study, 348 patients were included. The mean age of the patients was 46.3 years. Bilateral RRD was developed in 13.7% of the patients. It was found that total RRD in the first eye (*p*-value = 0.045), the presence of lattice degeneration in the first eye (*p*-value = 0.036), the presence of high-risk breaks (*p*-value = 0.0001) or lattice degeneration (*p*-value = 0.0004) in the fellow eye, the involvement of the inferior-nasal quadrant in the first eye (*p*-value = 0.043), and the presence of connective tissue diseases (*p*-value = 0.008) were significantly associated with the development of fellow-eye RRD. Performing prophylactic retinopexy was associated with a reduction in the incidence of fellow-eye RRD (with or without high-risk breaks) (*p*-value = 0.0001). It was not associated with a reduction in the risk of fellow-eye RRD in cases of lattice degeneration alone. **Conclusions**: Recognition of certain perioperative risk factors (such as high-risk retinal tears) during the presentation of first-eye RRD is crucial. Prophylactic laser retinopexy may have a critical role in preventing fellow-eye RRD. Patients’ awareness should be raised about the symptoms of RRD.

## 1. Introduction

Rhegmatogenous retinal detachment (RRD) is a vision-threatening retinal disorder, despite developed and successful treatment. Before the 20th century, unless the patient could compensate with one eye, RRD was suddenly blinding to the affected eye, and bilateral legal blinding due to fellow-eye RRD may have severely impaired vision with a complete loss of sight, loss of the ability to be independent and work, and adversely impacted the social, psychological, and economic burden of the individual and the healthcare system [1,2]. RRD is a well-recognized risk factor for RRD in the fellow eye. Everett described the term “fellow eye syndrome”, which refers to bilateral RRD and describes the symmetry of retinal pathologies between both eyes [3]. With the exclusion of trauma, all other predisposing factors that predispose an individual to RRD affect both eyes. These risk factors implicate high pathological myopia, the presence of high-risk retinal breaks, a family history of RRD, peripheral lattice degeneration, ocular surgery (mainly complicated surgery), and age-related symptomatic posterior vitreous detachment [1,4]. Moreover, in patients with unilateral RRD, 13% of fellow eyes (without RRD) were reported to have a visual acuity of 6/18 or worse [5]. Bilateral RRD has been reported widely in a range from 7% to 33% of patients, and, combining these results, the incidence of bilateral RRD is approximately 10% [1,6,7,8,9,10]. Accordingly, prophylactic management for the fellow eye can protect the patient from being legally blind and reduce the social, economic, and psychological burden of bilateral RRD on both the individual and the healthcare system.

The role of prophylactic laser retinopexy for the fellow eye without high-risk retinal lesions is still controversial, as the leading high-risk breaks often develop in areas of normal retina [11]. Prophylactic laser retinopexy for RRD is a fundamental emergency technique. Many techniques were developed for laser retinopexy; slit lamp-based laser retinopexy is the first line of prophylactic practice, and it is the most practiced method. Indirect-based laser retinopexy and cryotherapy for retinal breaks are specialized techniques. Without prophylactic laser, high-risk retinal lesions can develop into RRD in 30–50% of cases [7,12], which significantly diminishes to 2.1–8.8% following prophylactic laser retinopexy [13,14,15]. In addition, the RRD risk was reduced from 43% to 13% in fellow eyes with giant retinal tears with a prophylactic laser retinopexy [16]. On the other hand, even if high-risk breaks are well-secured, RRD in the fellow eye may be inevitable [11]. Instructing the patient regarding the presenting symptoms of RRD or high-risk breaks is crucial for the prevention of RRD, avoiding macular involvement, and preserving visual acuity. In this study, we investigate the demographic, clinical, and anatomical characteristics of RRD patients in a tertiary hospital in Jordan. Furthermore, this study aims to assess the factors that predispose the patients to bilateral RRD. Moreover, this study evaluates the role of prophylactic retinopexy in preventing fellow-eye RRD.

## 2. Materials and Methods

The primary outcome was to report the development and risks of RRD in the fellow eyes for patients who experienced RRD in the first eye. The risk factors expressed in the analysis were assessed at presentation in order to adjust the risk of fellow-eye RRD from the presentation. Secondary outcomes included the prophylactic effect of laser retinopexy for the fellow eye and the adjustment of a possible scoring system that may help in categorization of patients into high-risk, who need prophylactic retinopexy, and low-risk, who need observation only.

### 2.1. Patients and Data

This study was conducted in accordance with the ethical standards of the Declaration of Helsinki and its later amendment and obtained the ethical approval of the institutional review board (IRB) at the Jordan University of Science and Technology (JUST) and its affiliated tertiary hospital, King Abdullah University Hospital (KAUH). Retrospectively, we have involved all patients who underwent RRD repair through pars plana vitrectomy (PPV) at KAUH during the period of January 2015 to December 2023. A medical electronic database at KAUH was utilized to extract the data, which include general demographic characteristics and the medical history of the patients. Moreover, operative characteristics of RRD and its associated tears, along with other pathological retinal findings, were included. Furthermore, fellow-eye parameters (performing prophylactic retinopexy, the development of RRD, and the presence of retinal pathologies) were studied and investigated. The analyzed variables mostly pertained to pathologies of the first eye, as the aim of the study is to adjust and identify those who are at risk of bilateral RRD from the presentation.

Inclusion criteria comprised patients who underwent PPV for RRD and followed up at least 6 months after surgery. Exclusion criteria included patients who were treated for exudative retinal detachment, tractional retinal detachment, epiretinal membranes, idiopathic macular holes, and for drop nuclear material or drop intraocular lenses (IOL) after posterior segment-complicated cataract surgery. Furthermore, patients with macular degeneration, retinal vein occlusion, proliferative diabetic retinopathy, uveitis syndromes, and retinal dystrophies were excluded. Moreover, patients with giant retinal tears, retinal dialysis, choroidal detachment, retinoschisis-related RRD, or RRD repaired with scleral buckle were excluded. In addition, patients with insufficient data or with early loss of follow-up were also excluded.

Demographic data include the gender of the patients, the age at first presentation for RRD, and the laterality of the first eye of RRD. A comprehensive medical history was obtained and comprised the history of diabetes mellitus, hypertension, asthma, seizure disorders, and Marfan syndrome. In addition, the presence of any type of glaucoma before the development of RRD was investigated.

The retina was divided into four quadrants: superior-temporal, superior-nasal, inferior-temporal, and inferior-nasal. RRD was classified first according to the initial site of detachment (at presentation) regardless of the number of detached quadrants. It was classified into superior-based RRD, inferior-based RRD, total RRD (at presentation), and posterior pole detachment. Furthermore, the number of quadrants involved in the RRD was calculated. Macular status was also reported and divided into macula-on (where the macula was still attached at the time of operation) and macula-off (where the macula was detached at the time of operation). In cases of partial detachment of the macula, the macula is considered “on” if the fovea was attached and “off” if the fovea was detached. In these cases, the clinical attachment or detachment of the fovea was confirmed by optical coherence tomography (OCT).

Break locations were classified first as supertemporal, superanasal, inferotemporal, inferonasal, and posterior pole breaks. If a break was located on the horizontal line that connected the fovea, the break was classified as superior. In cases of more than one break, the location of the most important tear with more likelihood to initiate RRD was reported. The types of breaks that were involved in the analysis were horseshoe tears or operculated tears. Additional retinal pathologies were reported and investigated and comprised lattice degeneration, macular holes, non-diabetic vitreous hemorrhage, proliferative vitreoretinopathy (PVR; grade B or more), and insignificant myopic changes. Regarding bilateral RRD, all patients were assessed for the development of RRD in the follow-up and the duration between the first-eye RRD and fellow-eye RRD was measured. Furthermore, performing prophylactic retinopexy for the fellow eye was included for all patients (those with and without fellow-eye RRD). Moreover, the presence of retinal breaks (horseshoe and operculated) and lattice degenerations was allocated for all patients.

### 2.2. Diagnostic Settings of RRD

All patients underwent comprehensive ophthalmic examinations by vitreoretinal consultants or well-trained residents during the follow-up visits, including best-corrected visual acuity (BCVA), using a Snellen visual acuity chart by decimal unit. Then, the BCVA was converted to LogMAR visual acuity. For low visual acuities of counting fingers, hand motion, light perception, or “no light perception”, they were converted according to Schulze-Bonsel et al. [17]. These low visual acuity measures were expressed as LogMAR units of more than 2.0 LogMAR units. The Goldmann applanation tonometer was utilized to measure the intraocular pressure. Slit-lamp and indirect biomicroscopes were used to assess the lens status, anterior segment, and fundus conditions. The ocular parameters included the axial length (through applanation ultrasound measurement A-scan) and spherical equivalent (through autorefractor (Nidek ARK-510A)). Spectral domain-OCT (Retinascan RS-3000; NIDEK, Gamagori, Japan) was utilized to study the foveal status in cases of partially detached macula. In cases of media opacity, B-scan ultrasonography (NIDEK, Gamagori, Japan) was used to assess the status of the retina. The diagnosis of RRD, along with its extension, the number and location of breaks, the presence of PVR, and the presence of other findings, was established by the examination and agreement of the consultant vitreoretinal surgeon and well-trained residents.

### 2.3. Surgical Settings of RRD

Under general or retrobulbar anesthesia, all operations were performed by three consultant vitreoretinal surgeons who follow the same principle procedural guidelines. All operations were carried out using a 23 g vitrectomy system (Combined Wide-Field Elite Pack, Bausch, and Lomb). Three trocars were inserted 3.5 mm (in pseudophakic or aphakic) or 4 mm (in phakic) posterior to the surgical limbus, two of them temporally and one of them in the superior-nasal quadrant. A perfusion tube was placed into the infratemporal cannula, and the perfusion pressure was set at 30 mmHg. By setting the perfusion pressure at 30 mmHg and the maximum vacuum at 500 mmHg, core vitrectomy and complete peripheral vitreous shaving were performed at a cut rate of 5000 cuts/min (CPM). Retinal reattachment with perfluorocarbon liquid, endo-laser photocoagulation (around the breaks and 360° peripheral retina), air–perfluorocarbon exchange, and retinal tamponade were performed after that. Internal limiting membrane dye was injected in some cases. PVR, if present, was peeled using intraocular forceps. The decision of tamponades was individualized case by case and according to surgeon preference. Retinopexy for the fellow eye was performed in patients with or without retinal pathology to create adequate chorioretinal adhesions and to minimize the risk of fellow-eye RRD. Retinopexy was performed through laser photocoagulation with a slit-lamp laser using a contact lens (SuperQuad lens, Volk, Volk Optical, Inc, Mentor, OH, USA).

Laser photocoagulation was performed using a green argon laser through the ophthalmic pan-retinal photocoagulation device (Valon Lasers Oy, Merimiehenkuja 5, Vantaa, Finland). The wavelength of the laser was 532 nm, and the frequency of the mode laser was doubled Nd-YVO. In most cases, the prophylactic laser retinopexy was conducted around the high-risk lesions and in a 360-degree pattern by applying 2–3 lines of laser burns. During the follow-up, if the patients were found to have unsecured areas, additional laser retinopexy was applied. Laser retinopexy was performed mainly by well-trained senior residents.

### 2.4. Statistical Analysis

The IBM SPSS statistical package v.26 (Armonk, NY, USA) was utilized for the statistical analysis. Nominal categorical variables (such as gender or laterality) were presented as frequency (percentage). The normality of the continuous variables (such as age) was tested using the Kolmogorov–Smirnov test and expressed as mean ± standard error of the mean (SEM). The chi-square test was utilized to determine the statistical significance for categorical variables and the ANOVA test for continuous variables. A binary logistic regression analysis was used to investigate the independent factors affecting the development of bilateral RRD. ROC curves were utilized to assess the predictability of independent factors for the development of fellow-eye RRD. Kaplan–Meier and Cox regression tests were employed to assess the factor of variability of follow-up time on the development of fellow-eye RRD. A statistically significant result was considered if *p* ≤ 0.05.

## 3. Results

### 3.1. General Demographics and Characteristics of the Cohort

In this study, 348 patients were included, and the general characteristics were summarized in Table 1. Of those patients, 255 (73.3%) were males. The mean age of the patients was 46.3 years. The right eye was involved in 177 (50.3%) of the cases. The mean axial length of the eyes was 24.99 mm with a mean spherical equivalent of −2.43 diopter. Hypertension was reported in 92 (26.4%) of the patients, 61 (17.5%) of the patients had diabetes mellitus, 6 (1.7%) had seizure disorders, 8 (2.3%) had asthma, and 4 (1.1%) had Marfan syndrome. According to the pathology in the fellow eye at the time of presentation, 108 (31.0%) patients had retinal break while 76 (21.8%) had lattice degeneration. Moreover, 117 (33.6%) of the patients underwent prophylactic retinopexy in the fellow eye. RRD was developed in the fellow eye in 47 (13.7%) patients with a mean duration of development of the RRD from the first-eye RRD of 45.22 months.

### 3.2. Risk Factors in the First Eye Affecting the Development of Bilateral RRD

Total RRD in the first eye was significantly associated with a higher risk of development of fellow-eye RRD (21.7% of patients developed fellow RRD). Moreover, if the RRD in the first eye involved the inferior-nasal quadrant of the retina, it was related to an increase in the risk of fellow-eye RRD. The numbers and locations of the tears of the first eye had no significant relation to the development of fellow-eye RRD. This was also applied to the status of the macula. Out of the 98 patients with lattice degeneration in the first eye, 19 (19.4%) patients developed RRD in the fellow eye, which was significant.

### 3.3. Risk Factors in the Fellow Eye Affecting the Development of Bilateral RRD

The study revealed that there was no relation between the development of fellow-eye RRD and sex, laterality, ocular parameters, and previous ocular history. Regarding the comorbidities, the risk of development of fellow-eye RRD was significantly increased in patients with seizure disorders and Marfan syndrome. The presence of retinal tear (horse-shoe tear or operculated) and lattice degeneration in the fellow eye at presentation significantly increased the risk of the development of fellow-eye RRD. Performing prophylactic retinopexy for the fellow eye was effective in decreasing the chance of the development of fellow-eye RRD (6.0% of patients who underwent prophylactic retinopexy developed fellow-eye RRD in comparison with 17.3% who did not undergo it). Table 1 shows the factors affecting the development of fellow-eye RRD. It is important to emphasize that the risk factors studied in relation to the fellow eye were those factors that were presented during the first visit of the first-eye RRD.

### 3.4. Logistic Regression Analysis and Prediction Model for the Development of Fellow-Eye RRD

Binary logistic regression analysis was performed measuring factors that may affect the development of fellow-eye RRD. Factors that were significant in the univariate analyses were enrolled in the equation. It was revealed that performing prophylactic retinopexy and the presence of fellow-eye tears were independent factors affecting the development of fellow-eye RRD. Patients who did not receive retinopexy were at 4.3-fold risk of developing fellow-eye RRD. Furthermore, patients with fellow-eye tears were at a 3.7-fold chance of developing fellow-eye RRD. Table 2 clarifies the details of the binary logistic regression analysis. The results of the binary logistic regression analysis were consistent with the results of Table 3 in which cases of retinal tears and lattice degeneration were investigated separately regarding the effect of prophylactic retinopexy in preventing fellow-eye RRD in those lesions. It was demonstrated that cases of retinal tear alone or in combination with lattice degeneration were significantly related to the protective effects of prophylactic retinopexy. However, in cases of lattice degeneration alone, the prophylactic retinopexy had no role in preventing fellow-eye RRD.

**Figure 1:**
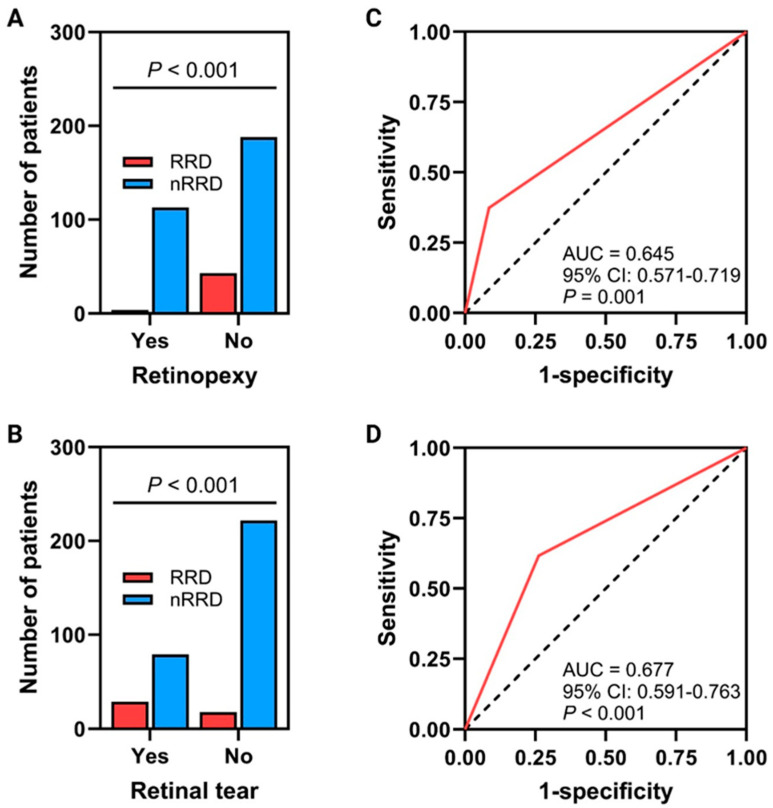
(**A**,**B**) Showed the relation between the presence of retinal tears and performing retinopexy and between the development of fellow-eye RRD. (**C**,**D**) ROC curves for the prediction of these relationships (the dotted black line represents the diagonal of no discrimination, the red line represents the tested variables).

Prediction probabilities were measured for every patient to predict the risk of developing fellow-eye RRD based on the characteristics of the fellow eye and on whether the patients performed prophylactic retinopexy or not. It was revealed that the prediction probabilities were significant and consistent with the logistic regression analysis. Significantly associated variables with the development of RRD on ROC curves include a history of retinopexy and retinal tears. Although solid associations were detected, the predictability of the variables visualized using ROC curves and multivariate logistic regression requires further studies to evaluate them since this work is not a predictability modeling investigation. The ROC curve of retinopexy has an area under the curve (AUC) of 0.645 (95% CI: 0.571–0.719, *p* = 0.001), while the ROC curve of retinal tear has an AUC of 0.677 (95% CI: 0.591–0.763, *p* < 0.001) Figure 1C,D.

Regarding the variability of follow-up intervals on the development of fellow-eye RRD, the Kaplan–Meier test was carried out to investigate the effect of the independent factors (as tear, retinopexy, total RRD of the first eye) on the time between first-eye and fellow-eye RRD, and it was revealed that neither factor was correlated with the time required for the development of fellow-eye RRD.

### 3.5. Performing Prophylactic Retinopexy on Patients Without Tears and a Proposed Scoring System

A two-step, two-layered chi-square test was run to measure the relationship and effectiveness of performing fellow-eye prophylactic retinopexy in patients without fellow-eye pathologies and for each pathology (tear, lattice) separately (Figure 1A,B and Table 3). It was shown that 108 patients had fellow-eye retinal tears; of those, 71 (65.7%) underwent prophylactic retinopexy, and 2 (2.8%) of them only developed fellow-eye RRD. On the other hand, among the 108, 37 (34.3%) of them did not perform prophylactic retinopexy, and 27 (73%) of them developed fellow-eye RRD (*p*-value < 0.05). Nevertheless, 240 patients did not present with fellow-eye tears; of those, 46 (19.2%) underwent prophylactic retinopexy, and 2 (4.3%) developed fellow-eye RRD. Moreover, 194 (80.8%) of them did not perform prophylactic retinopexy, and 16 (8.2%) of them developed fellow-eye RRD (*p*-value was 0.2). On the other hand, regarding lattice degeneration, 272 presented without lattice degeneration; of those, 68 (25%) performed prophylactic retinopexy, and 2 (2.9%) developed fellow-eye RRD. Furthermore, 204 (75%) did not perform prophylactic retinopexy and 27 (13.2%) developed fellow-eye RRD (*p* value was 0.01). Visual outcomes analysis was performed in fellow eyes with RRD versus fellow eyes without RRD in terms of BCVA. It was revealed that patients without fellow-eye RRD had a better BCVA of nearly 11 letters. Furthermore, there was no report of any side effects following prophylactic retinopexy. Based on these measures, and on the result that performing fellow-eye prophylactic retinopexy for patients without fellow-eye tears was not associated significantly with a reduction in the chance of developing RRD, but, on the other hand, given that 13 patients without retinopexy developed RRD, compared to the two patients with retinopexy that developed RRD, and based on the worse visual outcome of developing RRD for every single eye, we would recommend the following scoring system to predict the value of performing prophylactic retinopexy for patients with fellow-eye RRD. This scoring system is based on the findings and risk factors during presentation and on the clinical experience and requires further prospective trials to justify the system:

1- First-eye RRD (2 points)
2- Patients with fellow-eye tears (4 points)
3- Patients with fellow-eye lattice degeneration (3 points)
4- Patients younger than 40 years of age (2 points)
5- Patients with total RRD in the first eye (2 points)
6- Patients with syndromes at risk (Marfan, Stickler’s) (2 points)
7- Patients with complicated ocular surgery (2 points)

Any patient with six or more points, we recommend an absolute indication of performing prophylactic retinopexy. Patients with five points or less, we recommend measuring the risks and benefits of performing prophylactic retinopexy with a lower threshold for performing prophylactic retinopexy.

## 4. Discussion

This study was conducted in a Jordanian population with RRD and aimed to assess the presenting factors affecting the occurrence of fellow-eye RRD and the role of retinopexy in preventing fellow-eye RRD. To the best of our knowledge, this is the first study to be conducted in Jordan. It was found that total RRD in the first eye, the presence of lattice degeneration in the first eye, the presence of high-risk breaks or lattice degeneration in the fellow eye, and the presence of a history of seizure syndromes or connective tissue diseases were significantly associated with the development of fellow-eye RRD. Performing prophylactic retinopexy was associated with a reduction in the incidence of fellow-eye RRD, whether the fellow eye had high-risk breaks or not.

RRD is the separation of the neurosensory retina from the underlying retinal pigment epithelium. It is well-known that the forces of attachment of the neurosensory retina to retinal pigment epithelium are weak, and, once overwhelmed, the detachment can develop rapidly [12,18]. Universally, myopia and the presence of horseshoe tears are considered amongst the strongest and most significant risk factors contributing to the incidence of bilateral RRD [6,19,20]. In a study conducted by Moussa et al., significant risk factors associated with RRD included a younger age group, high myopia, male gender, and the presence of horseshoe tears [20]. It has been widely accepted that the characteristics and location of the RRD are dictated by the type and location of the underlying retinal breaks. This was described by Lincoff in what are known as “Lincoff’s rules” [21,22]. In 2018, David Wong cited six new rules that describe the locations of retinal breaks in RRDs that do not obey Lincoff’s rules. He described that a retinal break in the superotemporal quadrant would result in a subtotal RRD, higher on the temporal side and bullous inferiorly (rule 1). A retinal break in the same location could result in an acute bullous superior RRD overhanging the posterior pole and macula (rule 2). The break is usually located in areas of thin retina in the detached retina (rule 3). In a fundus-obscuring vitreous hemorrhage, multiple retinal breaks should be suspected (rule 4). In the case of RRD involving the posterior retina with limited extension inferiorly and peripherally, the primary break is usually located at the posterior pole (rule 5). Finally, in inferior bullous RRD, the retinal breaks should be on the concave as opposed to the convex side (rule 6) [22].

RRD in one eye is a well-known risk for the development of RRD in the fellow eye. Although the incidence of bilateral RRD is not precisely known, the incidence of fellow-eye RRD is approximately 10% [4,8,11,14]. A large study was conducted by Radeck et al. on 5791 eyes with RRD, and they found that a total of 300 patients (5%) had bilateral RRD [1]. The interval between initial and fellow-eye RRD was 2.6 years [1]. According to their study, 220 (of the bilateral cases) patients were male (73%), and the mean age at initial RRD was 55 years [1]. About two-thirds of the first RRD eyes were phakic. The BEAVRS and EURETINA VR RD Outcomes Group found that the risk for fellow-eye RRD decreased linearly with age [23]. A multicenter study by Xu et al. revealed a mean age of 57 years, and 55% were pseudophakic in the first eye and 65% were pseudophakic in the second eye [24]. The Scottish retinal detachment study conducted by Mitry et al. showed that the bilateral RRD group was similar in age and gender to the unilateral group [5]. In this study, the mean age for patients with bilateral RRD was significantly younger than patients with unilateral RRD. In addition, males were more commonly affected in both unilateral and bilateral RRDs. The gender discrepancy could be explained by the fact that men have a larger axial length, and the variation in the size of the vitreous base could be explanatory [25,26]. Moreover, men are prone to more trauma, such as head contusions and ocular injury [27].

Regarding the anatomical and visual discrepancy between cases of unilateral versus bilateral RRD, Walia et al. reported symptoms for much longer duration, a higher rate of macula-off RRD, and worse visual acuity in the first eye compared to the second eye in bilateral RRD [28]. Similarly, Radech et al. reported that in the first eye, more patients had macular involvement, worse visual acuity, and more quadrants involved [1]. Furthermore, Xu et al. revealed that the presenting symptoms were 5.9 versus 7.5 days, involvement of the macula was 34% versus 56%, and a visual acuity at presentation of 20/62 in the second eye versus 20/149 in the first eye [24]. Mitry et al. reported attached macula in the second eye in 63% compared to 44% in the unilateral group; duration of symptoms, 13 versus 28 days; in the fellow eye compared to unilateral RD [5]. In this study, bilateral RRD was associated with total RRD in the first eye, and the patients tended to perform better with fellow-eye RRD. The longer the duration of RRD, the more it was a cause for the formation of PVR, which is a major pathology that pertains to the failure of RRD surgery [29,30,31].

In unilateral RRD, lattice degeneration has been reported in 9.2–35% of fellow eyes [4,6,9,32] and RRD develops in only 0.7% of these patients [33]. On the other hand, the risk of RRD due to lattice degeneration in the fellow eye was 5.1% [7]. This risk increases to 15% of cases at 10 years [7]. The risk of RRD as a result of lattice degeneration was reported to be 5.1% in eyes without prophylactic treatment versus 1.8% in fellow eyes prophylactically treated [8]. However, prophylactic treatment has not been shown to reduce the risk of RRD in cases of extensive lattice degeneration or high myopia [8]. Nevertheless, lattice RRD would be prevented in only 3% of patients treated in the fellow eye in patients with less than 6 D of myopia and less than 6 clock-hours of degeneration [8]. In this study, lattice degeneration was reported in about 28% of patients in the first eye of RRD and was found in about 21% of patients in the fellow eye. It was associated with fellow-eye RRD in 23.7% of patients (out of the 21%). The risk of fellow-eye RRD due to lattice was investigated in this study; it was shown that prophylactic retinopexy had no protective role in cases of lattice degeneration alone (without high-risk retinal tear), which is consistent with previous works [8]. On the other hand, cases of lattice degeneration combined with retinal tears would be secured effectively by prophylactic retinopexy.

Prophylactic laser retinopexy of high-risk lesions to prevent the development of RRD remains controversial [4,9,33,34]. Avitabile et al. reported in their study a reduction in the rate of bilateral RRD from 13.4% to 1.2% after cryotherapy, laser prophylactic retinopexy, or scleral buckle in 305 fellow eyes with high-risk retinal lesions. None of the fellow eyes without high-risk lesions developed RRD over the follow-up period [35]. Morris et al. investigated the results of observation, focal retinopexy, and 360-degree retinopexy on 269 pseudophakic fellow eyes [2]. They revealed that 33 of 175 untreated fellow eyes (19%) experienced RRD, 5 of 22 focally treated fellow eyes (23%) experienced RRD, and only one of 72 fellow eyes (1.4%) receiving 360-degree prophylaxis experienced RRD in a follow-up period averaging 5 years [2]. On the other hand, Folk and Burton supported the evidence of avoiding the prophylactic measures because they found no significant difference between the clinical characteristics of the two cohorts [8]. In this study cohort, we offered many patients 360-degree prophylactic retinopexy for the fellow eye, and we found that 7 of 117 (6%) that received prophylactic retinopexy developed fellow-eye RRD in comparison to the 40 of 231 (17.3%) untreated fellow eyes that developed RRD. Moreover, in patients who presented with a normal retina in the fellow eye (no high-risk breaks), 13 of 194 un-secured fellow eyes developed RRD in comparison to the 5 of 46 secured fellow eyes that developed RRD, which was not significant.

Even if the high-risk lesions are secured with a laser, RRD in the fellow eye may be unavoidable [4,11,36]. Retinal breaks with following RRD in the secured fellow eyes can also develop near (anterior or posterior) areas of prophylactic treatment in around 20% of cases [11]. Therefore, the safety and efficacy of prophylactic treatment remain controversial. Side effects for prophylactic laser retinopexy include vitreoretinal traction, epiretinal membrane formation, vitreous hemorrhage, retinal breaks, or even detachment [4]. In this study, in patients with high-risk retinal breaks in the fellow eye, 2 of 71 (2.8%) of secured fellow eyes with retinopexy developed RRD in comparison to the 27 of 37 (73%) of untreated fellow eyes that developed RRD. Moreover, no patient in the treated group experienced side effects of prophylactic laser retinopexy. Accordingly, due to the debate regarding the efficacy of prophylactic laser retinopexy, especially in those without predisposition, we have proposed a scoring system that may predict the possibility of developing RRD in the fellow eye; this scoring system is just a theory and needs to be justified in prospective clinical trials.

This study is not without limitations. First, the retrospective nature of the study limits its validity in relation to the results generated. Second, the deficient data regarding some patients is another limitation. Third, only horseshoe breaks and lattice degeneration were included in the fellow eye analysis as predisposing lesions; many other lesions may contribute to the development of fellow-eye RRD. Four, only the presenting risk factors were included in the analysis; factors that may develop on following the patient were not included. Finally, the variability in the follow-up time of the patients may influence the development of fellow-eye RRD.

## 5. Conclusions

In conclusion, the definitive study regarding the efficacy of prophylactic laser retinopexy for the prevention of RRD in the fellow eye has not yet been published. Indications for prophylactic laser retinopexy have not been uniform among existing studies. The mode of treatment, either argon laser or cryotherapy, also varied among studies. Individually comparing RRD rates of different pathologies may demonstrate which retinal lesions are more responsive to prophylactic treatment. This study reported a higher rate of bilateral RRD among patients with total RRD in the first eye, patients with high-risk lesions in the fellow eye, patients with connective tissue disorders, and in younger patients. Patients presented with fellow-eye high-risk retinal tears may have the most benefit from prophylactic retinopexy. Prophylactic retinopexy may have no role in patients with lattice degeneration alone in the fellow eye. A more convincing study could entail a prospective, randomized design among patients with similar predisposing lesions.

## Figures and Tables

**Table 1:** Factors affecting the development of fellow-eye RRD.

Variables		Number (Percentage) or Mean ± SEM		
	Overall Cohort (*n* = 348)	Fellow-Eye RRD (*n* = 47)	No Fellow-Eye RRD (*n* = 301)	*p*-Value
**Sex**				
Male	255 (73.3)	38 (14.9)	217 (85.1)	NS
Female	93 (26.7)	9 (9.7)	84 (90.3)	
**Age (years)**	46.3 ± 1.0	38.2 ± 2.9	47.6 ± 1.0	0.002
**Laterality of the first eye**				
Right eye	177 (50.9)	23 (48.9)	154 (51.2)	NS
Left eye	171 (49.1)	24 (51.1)	147 (48.8)	
**Ocular parameters of both eyes**				
Axial length of the first eye (mm)	24.99 ± 0.14	25.52 ± 0.5	24.94 ± 0.1	NS
Axial length of the fellow eye (mm)	24.81 ± 0.2	25.16 ± 0.3	24.78 ± 0.2	NS
Spherical equivalent of the first eye (diopter)	−2.43 ± 0.82	−2.83 ± 0.8	−2.40 ± 0.8	NS
Spherical equivalent of the fellow eye (diopter)	−2.39 ± 0.6	−2.75 ± 0.7	−2.34 ± 0.6	NS
**Medical history**				
**Diabetes mellitus**	61 (17.5)	8 (13.1)	53 (86.9)	NS
**Hypertension**	92 (26.4)	8 (8.7)	84 (91.3)	NS
**Cognitive impairment**	6 (1.7)	3 (50.0)	3 (50.0)	0.034
**Marfan syndrome**	4 (1.1)	3 (75.0)	1 (25.0)	0.008
**Asthma**	8 (2.3)	1 (12.5)	7 (87.5)	NS
Type of RRD at presentation of the first eye * (*n* = 290)				
Superior-based RRD	145 (50.0)	13 (9.0)	132 (91.0)	
Inferior-based RRD	75 (25.9)	7 (9.3)	68 (90.7)	
Total RRD	69 (23.8)	15 (21.7) ↑↑	54 (78.3)	0.045
Posterior pole detachment	1 (0.3)	0 (0.0)	1 (100.0)	
Number of involved quadrants of the first eye * (*n* = 276)				
One quadrant	16 (5.8)	1 (6.3)	15 (93.7)	
Two quadrants	134 (48.6)	14 (10.4)	120 (89.6)	NS
Three quadrants	47 (17.0)	3 (6.4)	44 (93.6)	
Four quadrants	79 (28.6)	15 (19.0) ↑	64 (81.0)	
Involvement of each quadrant separately (of the first eye) * (*n* = 231)				
**Superior-temporal quadrant**	189 (81.8)	23 (12.2)	166 (87.8)	NS
**Superior-nasal quadrant**	140 (60.6)	20 (14.3)	120 (85.7)	NS
**Inferior-temporal quadrant**	181 (80.8)	24 (13.3)	157 (86.7)	NS
**Inferior-nasal quadrant**	135 (58.4)	21 (15.6)	114 (84.4)	0.043
Macular status of the first eye * (*n* = 296)				
Macula on	57 (19.3)	5 (8.8)	52 (91.2)	NS
Macula off	239 (80.7)	31 (13.0)	208 (87.0)	
Location of the main tear according to quadrants of the first eye * (*n* = 258)				
Superior-temporal quadrant	143 (55.4)	15 (10.5)	128 (89.5)	
Superior-nasal quadrant	42 (16.3)	6 (14.3)	36 (85.7)	
Inferior-temporal quadrant	37 (14.3)	1 (2.7)	36 (97.3)	NS
Inferior-nasal quadrant	31 (12.0)	2 (6.5)	29 (93.5)	
Macular hole	5 (1.9)	1 (20.0)	4 (80.0)	
The presence of other retinal pathologies of the first eye * (*n* = 348)				
**Lattice degeneration**	98 (28.2)	19 (19.4)	79 (80.6)	0.036
**Macular hole**	13 (3.7)	1 (7.7)	12 (92.3)	NS
**VH**	23 (6.6)	3 (13.0)	20 (87.0)	NS
**PVR**	109 (31.3)	18 (16.5)	91 (83.5)	NS
**The presence of retinal pathology in the fellow eye**				
**Retinal tear (horse-shoe tear)**	108 (31.0)	29 (26.9)	79 (73.1)	0.0001
**Lattice degeneration**	76 (21.8)	18 (23.7)	58 (76.3)	0.0004
**Performing prophylactic retinopexy for the fellow eye**				
Yes	117 (33.6)	7 (6.0)	110 (94.0)	0.0001
No	231 (66.4)	40 (17.3)	191 (82.7)	

Abbreviations: SEM: standard error; VH: vitreous hemorrhage; RRD: rhegmatogenous retinal detachment; NS: not significant; PVR: proliferative vitreoretinopathy. * The sample size for these variables is indicated separately. ↑: increase, ↑↑: significant increase.

**Table 2:** Binary logistic regression analysis of the factors affecting the development of fellow-eye RRD.

Variables	B-value	SEM	*p*-Value	Exp(B)	95% CI	
**Age**	0.001	0.015	0.927	1.001	0.973	1.030
**Cognitive impairment**	−2.085	1.452	0.151	0.124	0.007	2.139
**Marfan syndrome**	−3.414	5.624	0.544	0.033	0.000	2015.71
**Type of RD at presentation of the first eye**						
Superior-based RRD			0.496			
Inferior-based RRD	−1.142	1.028	0.266	0.319	0.043	2.393
Total RRD	−1.170	0.844	0.165	0.310	0.059	1.621
Posterior pole detachment	−19.11	40,192.97	1.000	0.000	0.000	.
**Tears of the fellow eye**	−3.749	0.735	0.0001	0.024	0.006	0.100
**Lattice degeneration of the fellow eye**	−0.073	0.824	0.929	0.930	0.185	4.672
**Prophylactic retinopexy for the fellow eye**	4.246	0.962	0.0001	69.832	10.598	460.154
**Constant**	3.039	5.809	0.601	20.893		

**Table 3:** Detailed description of the relation between prophylactic retinopexy and the development of fellow-eye RRD versus type of pathology.

Prophylactic Retinopexy		Number (Percentage)		
	Retinal Lesion	Fellow-Eye RRD	No Fellow-Eye RRD	*p*-Value
Prophylactic retinopexy				
Yes	All cases of retinal tears (*n* = 108)	2 (2.8)	69 (97.2)	0.0001
No		27 (73.0)	10 (27.0)	
Prophylactic retinopexy				
Yes	Retinal tears alone * (*n* = 57)	2 (5.9)	32 (94.1)	0.0001
No		13 (56.5)	10 (43.5)	
Prophylactic retinopexy				
Yes	All cases of lattice degeneration (*n* = 76)	2 (4.1)	47 (95.9)	0.0001
No		16 (59.3)	11 (40.7)	
Prophylactic retinopexy				
Yes	Lattice degeneration alone ** (*n* = 25)	2 (16.7)	10 (83.3)	NS
No		2 (15.4)	11 (84.6)	
Prophylactic retinopexy				
Yes	Combination of retinal tears and lattice degeneration (*n* = 51)	0 (0.0)	37 (100.0)	0.00001
No		14 (100.0)	0 (0.0)	

Abbreviations: RRD: rhegmatogenous retinal detachment; NS: not significant. * Included cases of retina with tears alone (without lattice degeneration). ** Included cases of retina with lattice degeneration alone (without tears).

## Data Availability

The datasets generated and analyzed during the current study are available from the corresponding author.
